# Determinants of psychoactive substances use among Woldia University students in Northeastern Ethiopia

**DOI:** 10.1186/s13104-017-2763-x

**Published:** 2017-09-05

**Authors:** Ashete Adere, Nigus Bililign Yimer, Henok Kumsa, Misgan Legesse Liben

**Affiliations:** 1 Department of Midwifery, College of Health Sciences, Woldia University, Woldia, Amhara Ethiopia; 2 Department of Public Health, College of Medical and Health Sciences, Samara University, P.O.Box 132, Samara, Afar Ethiopia

**Keywords:** Psychoactive substances, Khat, Alcohol, Cigarette, University students, Woldia

## Abstract

**Background:**

Psychoactive substance use has become a major public health problem among students in Ethiopian universities. Hence, this study aimed to investigate the magnitude and determinants of psychoactive substances use (khat chewing, alcohol drinking and cigarette smoking) among undergraduate students of Woldia University, Ethiopia.

**Methods:**

Institution based quantitative cross-sectional study was employed on Woldia University students in April 2015. 730 students were included in the study. Data were collected using self-administered questionnaires. EpiData version 3.02 was used to enter data. Then, data were exported to SPSS version 20 for analysis.

**Results:**

The lifetime prevalence of alcohol drinking, khat chewing and cigarette smoking among the study students were 33.1, 13 and 7.9%, respectively. Likewise, the current prevalence of alcohol drinking, khat chewing and cigarette smoking was 27.9, 10.4 and 6.4%, respectively. More than half of the surveyed students (59.1%) were introduced to psychoactive substance use by peer pressure. About 66% of the study participants believed that psychoactive substances are important for relaxation, and 19% to relief from stress. Students who were Muslims [adjusted odds ratio (AOR) 3.74, 95% CI (1.57, 8.91)], Oromo ethnic group [AOR 2.63, 95% CI (1.19, 5.81)], ever drunk alcohol [AOR 6.32, 95% CI (2.96, 13.48)] and ever smoked cigarette [AOR: 9.16, 95% CI (4.33, 19.38)] were positively associated with khat chewing. Furthermore, pocket money and ever khat chewing were associated with alcohol drinking and cigarette smoking.

**Conclusion:**

This study showed that psychoactive substances use is somewhat prevalent among students in Woldia University. Hence, support of religious institutions in providing education aimed at preventing substance use, establishing and strengthening peer educators in the university are important interventions to tackle psychoactive substances use.

## Background

Psychoactive substance is a substance that acts mainly on the central nervous system where it alters brain function. It can result short-term changes in perception, mood, consciousness and behavior [1]. Worldwide approximately two billion people drink alcohol, in which alcohol contributes 4% of the global burden of disease [2]. In most parts of Africa, health and social problems associated with alcohol drinking are increasing. In sub-Saharan Africa an estimated 1.8% of the disease burden is attributable to alcohol consumption [3, 4].

Psychoactive substance use has become a major public health problem in Ethiopia. The overall prevalence of khat chewing in Ethiopia is 15.3%. Khat chewing was highly prevalent (53.2%) in Harari regional state. This was followed by Dire Dawa (44.9%), Oromia (26.4%), Somali (26.0%) and Tigray regional state (1.1%) [5]. Cross sectional studies in different parts of Ethiopia showed that khat chewing was associated with mental distress, risky sexual behavior and diminished academic performance [6–8]. Poorer academic performance associated with khat chewing was also documented among students in Saudi Arabia [9].

Cigarette smoking is spreading rapidly in developing countries, including Ethiopia. Currently, in developing countries 50% of men and 9% of women smoke cigarette, as compared with 35% of men and 22% of women in developed countries [10]. In Ethiopia, 4% of youths and 6.3% of individuals in age groups of 25–29 years practice cigarette smoking. Moreover, 45% of women and 53% of men ever drank alcohol. Alcohol drinking is highest among individuals having preparatory and more educational status [11].

Students in higher institution are at risk of using alcohol, khat and tobacco. Students entering the University had the opportunities of freedom from family control and peer pressure to use psychoactive substances. Therefore, psychoactive substance use is becoming a common practice among adolescents and youths in general, and university students in particular [5, 12–15].

Despite of this fact, few studies have been conducted on the factors associated with consumption of psychoactive substances among students in Ethiopian universities. Hence, this study was conducted to investigate the magnitude and determinants of psychoactive substances use among undergraduate students of Woldia University in Ethiopia.

## Methods

### Study setting and design

Institution based quantitative cross-sectional study was employed on Woldia University students during April 2015. Woldia University is one of the third generation higher education institutions in Ethiopia; and it has been established in 2011. The university is located in Woldia town of Amhara Regional State at about 521 km northeast of Addis Ababa (the capital city of Ethiopia). Woldia University has two campuses: Mersa and Woldia. The University consists of 8 faculties with 8695 regular undergraduate students.

### Sample size and sampling procedure

A sample size of 730 was considered using single population proportion formula with the following assumptions; prevalence of alcohol drinking 34.5% in the last 12 months [13], $z\frac{\alpha }{2}$ = critical value for normal distribution at 95% confidence level which equals to 1.96 (Z value at alpha 0.05), d = 0.05 (an absolute precision), 5% estimated non-response rate and design effect of two.

In this study all regular undergraduate students attending at Woldia University were included as study population. Multistage sampling technique was used to select the study subjects. First, of the eight faculties in the university, four faculties were selected by lottery. In the second stage, selected faculties were further stratified by sex, assuming that their sex would affect psychoactive substance use. Then, the sample size was proportionally allocated to randomly selected faculties based on number of students in each sex. Finally, the study units in each stratum were selected by lottery using students’ identification number. Study participants who were seriously ill, unable to communicate and absent during the study period were excluded from the study.

### Study variables

The dependent variables were alcohol drinking, khat chewing and cigarette smoking. Lifetime magnitude of substance use is the proportion of students who had ever reported substance use in their lifetime. Current magnitude of substance use is the proportion of students who reported substance use within 30 days preceding the study. The independent variables were socio-demographic characteristics (age, sex, religion and ethnicity), average monthly pocket money, year and faculty of study.

### Data collection process and instrument

Data were collected using self-administered questionnaire. The questionnaire was prepared by reviewing related literatures [12–18]. The questionnaire was prepared first in English and translated to Amharic (the Ethiopian national language), then back to English to check for consistency. The questionnaire was pre-tested on 5% of the sample size at Northeast Health Science College, located in Woldia town. Then, Amharic version of the questionnaire was used to collect the data. Three university lecturers supervised the data collection process. Confidentiality of responses was maintained throughout the study.

### Data processing and analysis

The data were checked for completeness and consistencies. EpiData version 3.02 was used to enter data. Then, data were exported to SPSS version 20 for analysis.

First, crude odds ratio (COR) with 95% confidence interval was estimated in the univariable logistic regression analysis. Then, to identify independent factors associated with substance use, variables with p value <0.25 in the univariable logistic regression analysis were considered in the multivariable logistic regression analysis [19, 20]. The Hosmer–Lemeshow goodness-of-fit with enter procedure was used to test for model fitness. Finally, adjusted odds ratio (AOR) with 95% confidence interval was estimated to assess the strength of the association, and variables with p value <0.05 were considered as significant and independent correlates.

## Results

### Characteristics of the study participants

Of 730 study students, 655 students completed the questionnaires. This results a response rate of 89.7%. Four hundred fifty-four (69.3%) of the study students were males. The mean age of the students was 20.74 (SD ± 1.36) years. Majority of the students (60.9%) were ethnically Amhara and 477 (72.8%) were Ethiopian Orthodox Christians (Table 1).

**Table 1:** Socio-demographic characteristics of Woldia University students (n = 655), Ethiopia, 2015

Variables	Number	%
Age		
18–19	111	16.9
20–24	539	82.3
≥25	5	0.8
Sex		
Male	454	69.3
Female	201	30.7
Ethnicity		
Amhara	399	60.9
Oromo	94	14.3
SNNP^a^	87	13.3
Tigray	75	11.5
Religion		
Orthodox	477	72.8
Muslims	95	14.5
Protestant	69	10.5
Others^b^	14	2.2
Year of study		
First year	226	34.5
Second year	285	43.5
Third year	113	17.3
Fourth year	31	4.7
Faculty		
Health science	45	6.9
Social science	131	20.0
Natural science	164	25.0
Technology	315	48.1
Average monthly pocket money (ETB)		
<250	174	26.6
250–499	286	43.6
≥500	195	29.8

*ETB* Ethiopian Birr

^a^Southern nations, nationalities and peoples

^b^Catholic and traditional

### Types of psychoactive substances

Overall, 242 (36.9%) of the study students had used psychoactive substances in their lifetime, and 206 (31.5%) were current users. The lifetime magnitude of khat chewing was 13%, alcohol drinking (33.1%) and cigarette smoking was reported by 7.9% of the study students. Likewise, the current magnitude of khat chewing, alcohol drinking and cigarette smoking was 10.4, 27.9 and 6.4%, respectively (Table 2). Furthermore, 11 (1.7%) of the study students had used cocaine, 10 (1.5%) cannabis, and 7 (1.1%) diazepam.

**Table 2:** Lifetime and current magnitude of psychoactive substance use among Woldia University students by sex, 2015

Psychoactive substance use	Sex		Total, n (%)
	Male n (%)	Female n (%)	
Ever used			
Yes	192 (42.3)	50 (24.9)	242 (36.9)
No	262 (57.7)	151 (75.1)	413 (63.1)
Substance use in the last 12 months			
Yes	174 (38.3)	50 (24.9)	234 (35.7)
No	280 (61.7)	151 (75.1)	421 (64.3)
Current users			
Yes	162 (35.7)	44 (21.9)	206 (31.5)
No	292 (64.3)	157 (78.1)	449 (68.5)
Khat chewing			
Ever chewed			
Yes	74 (16.3)	11 (5.5)	85 (13.0)
No	380 (83.7)	190 (94.5)	570 (87.0)
Chewed in the last 12 months			
Yes	63 (13.9)	9 (4.5)	72 (11.0)
No	391 (86.1)	192 (95.5)	583 (89.0)
Current chewers			
Yes	59 (13.0)	9 (4.5)	68 (10.4)
No	395 (87.0)	192 (95.5)	587 (89.6)
Alcohol drinking			
Ever drunk			
Yes	171 (37.7)	46 (22.9)	217 (33.1)
No	283 (62.3)	155 (77.1)	438 (66.9)
Drunk in the last 12 months			
Yes	167 (36.8)	46 (22.9)	213 (32.5)
No	287 (63.2)	155 (77.1)	442 (67.5)
Current drinkers			
Yes	143 (31.5)	40 (19.9)	183 (27.9)
No	311 (68.5)	161 (80.1)	472 (72.1)
Cigarette smoking			
Ever smoke			
Yes	46 (10.1)	6 (3.0)	52 (7.9)
No	408 (89.9)	195 (97.0)	603 (92.1)
Smoke in the last 12 months			
Yes	39 (8.6)	6 (3.0)	45 (6.9)
No	415 (91.4)	195 (97.0)	610 (93.1)
Current smokers			
Yes	36 (7.9)	6 (3.0)	42 (6.4)
No	417 (92.1)	195 (97.0)	612 (93.6)

Thirty-one (12.8%) of the study participants started using psychoactive substances while they were at elementary school, 34 (14%) at secondary, 72 (29.8%) at preparatory and 105 (43.4%) of the students at University level. One hundred forty- three students (59.1%) were introduced to psychoactive substances use by peer friends, 54 (22.3%) by themselves, 30 (12.4%) by the surrounding community, and 15 (6.2%) by family members. From 242 students who reported ever use of psychoactive substances, 159 (65.7%) reported substance use to relax with friends, and 45 (18.6%) students believed that psychoactive substances are important to get relief from stress. Fifteen students (6.2%) reported use of substances to keep alert while reading and 45 (18.6%) for religious purposes.

### Factors associated with psychoactive substances use

In the multivariable analysis sex, religion, ethnicity, year of study, ever drunk alcohol, ever smoked cigarette and average monthly pocket money were significantly associated with khat chewing in the last 12 months. Khat chewing was practiced more by males as compared to females [AOR 2.45, 95% CI 1.06, 5.66)]. Students who were Muslims were about four times [AOR 3.74, 95% CI (1.57, 8.91)] more likely to chew khat as compared to students who were Ethiopian Orthodox Christians. Students who had ever drunk alcohol [AOR 6.32, 95%CI (2.96, 13.48)] and ever smoked cigarette [AOR 9.16, 95% CI (4.33, 19.38)] were more likely to chew khat as compared to students who had never drunk and smoked cigarette, respectively (Table 3).

**Table 3:** Logistic regression analysis showing factors associated with khat chewing within the last 12 months among Woldia University students, 2015

Variables	Khat chewing		COR (95% CI)	AOR (95% CI)
	Yes	No		
Sex				
Male	63	391	3.44 (1.67, 7.06)*	2.45 (1.06, 5.66)*
Female	9	192	1	1
Religion				
Orthodox	46	431	1.00	1.00
Muslims	16	79	1.90 (1.02, 3.52)*	3.72 (1.56, 8.87)*
Others^a^	10	73	0.89(0.37, 2.18)	0.99 (0.36, 2.77)
Ethnicity				
Amhara	30	369	1.00	1.00
Oromo	20	74	3.32 (1.79, 6.17)**	2.69 (1.21, 6.01)*
SNNP^b^	13	74	2.16 (1.08, 4.34)*	2.03 (0.77, 5.32)
Tigray	9	66	1.68 (0.76, 3.69)	1.88 (0.70, 5.09)
Year of study				
1st year	17	209	1.00	1.00
2nd year	32	253	1.56 (0.84, 2.88)	1.57 (0.73, 3.39)
3rd year	18	95	2.33 (1.15, 4.72)*	2.52 (1.06, 5.98)*
4th year	5	26	2.36 (0.81, 6.94)	3.03 (0.78, 11.82)
Ever drunk alcohol				
Yes	54	163	7.73 (4.40, 13.58)**	6.37 (2.97, 13.67)**
No	18	420	1.00	1.00
Ever smoked cigarette				
Yes	32	20	22.52 (11.83, 42.89)**	9.16 (4.33, 19.38)**
No	40	563	1.00	1.00
Average monthly pocket money (ETB)				
<250	5	169	1.00	1.00
250–499	30	256	3.96 (1.51, 10.41)*	3.37 (1.14, 9.98)*
≥500	37	158	7.92 (3.04, 20.65)**	5.28 (1.77, 15.77)*

Hosmer–Lemeshow goodness-of-fit = 0.271

*ETB* Ethiopian Birr, *COR* crude odds ratio, *AOR* adjusted odds ratio, *CI* confidence interval

^*^Statistically significant variables at p < 0.05

^**^Statistically significant variables at p < 0.001

^a^Protestant, Catholic and traditional

^b^Southern nations, nationalities and peoples

Year of study was not associated with alcohol drinking and cigarette smoking in the last 12 months (p > 0.25). Multivariable logistic regression analysis revealed that sex, religion, ethnicity, ever khat chewing and average monthly pocket money were associated with alcohol drinking in the last 12 months (Table 4).

**Table 4:** Logistic regression analysis showing factors associated with alcohol drinking within the last 12 months among Woldia University students, 2015

Variables	Alcohol drinking		COR (95% CI)	AOR (95% CI)
	Yes	No		
Sex				
Male	167	287	1.96 (1.34, 2.87)*	2.25 (1.46, 3.45)**
Female	46	155	1.00	1.00
Religion				
Orthodox	179	298	1.00	1.00
Muslims	11	84	0.22 (0.11, 0.42)**	0.14 (0.07, 0.29)**
Protestant	15	54	0.46 (0.25, 0.84)*	0.54 (0.26, 1.13)
Others^a^	8	6	2.22 (0.76, 6.50)	1.93 (0.55, 6.8)
Ethnicity				
Amhara	134	265	1.00	1.00
Oromo	31	63	0.97 (0.60, 1.57)	0.75 (0.42, 1.34)
SNNP^b^	20	67	0.59 (0.34, 1.01)*	0.48 (0.24, 0.97)*
Tigray	28	47	1.18 (0.71, 1.97)	1.04 (0.59, 1.81)
Ever chewed khat				
Yes	60	25	6.54 (3.96, 10.81)**	6.97 (3.88, 12.55)**
No	153	417	1.00	1.00
Average monthly pocket money (ETB)				
<250	43	131	1.00	1.00
250–499	91	195	1.42 (0.93, 2.17)	1.59 (0.99, 2.55)
≥500	79	116	2.08 (1.33, 3.25)*	2.37 (1.42, 3.96)*

Hosmer–Lemeshow goodness-of-fit = 0.882

*ETB* Ethiopian Birr, *COR* crude odds ratio, *AOR* adjusted odds ratio, *CI* Confidence Interval

^*^Statistically significant variables at p < 0.05

^**^Statistically significant variables at p < 0.001

^a^Catholic and traditional

^b^Southern nations, nationalities and peoples

Furthermore, average monthly pocket money, ever khat chewing and ever alcohol drinking were positive correlates of cigarette smoking in the multivariable logistic regression analysis at p < 0.05 (Table 5).

**Table 5:** Logistic regression analysis showing factors associated with cigarette smoking within the last 12 months among Woldia University students, 2015

Variables	Cigarette smoking		COR (95% CI)	AOR (95% CI)
	Yes	No		
Sex				
Male	39	415	3.05 (1.27, 7.34)*	1.64 (0.58, 4.62)
Female	6	195	1.00	1.00
Religion				
Orthodox	32	445	1.00	1.00
Muslims	6	89	0.94 (0.38, 2.31)	1.60 (0.47, 5.53)
Others^a^	7	76	1.28 (0.55, 3.01)	0.48 (0.09, 2.29)
Ethnicity				
Amhara	22	377	1.00	1.00
Oromo	11	83	2.27 (1.06, 4.87)*	1.26 (0.48, 3.31)
SNNP^b^	7	80	1.50 (0.62, 3.63)	0.83 (0.25, 2.79)
Tigray	5	70	1.20 (0.45, 3.34)	0.93 (0.28, 3.10)
Ever chewed khat				
Yes	32	53	25.87 (12.8, 52.28)**	10.4 (4.76, 22.74)**
No	13	557	1.00	1.00
Ever drunk alcohol				
Yes	40	177	19.57 (7.6, 50.4)**	10.8 (3.55, 32.96)*
No	5	433	1.00	1.00
Average monthly pocket money (ETB)				
<250	4	170	1.00	1.00
250–499	16	270	2.52 (0.83, 7.66)	1.51 (0.43, 5.29)
≥500	25	170	6.25 (2.13, 18.34)*	3.89 (1.17, 11.38)*

Hosmer–Lemeshow goodness-of-fit = 0.892

*ETB* Ethiopian Birr, *COR* crude odds ratio, *AOR* adjusted odds ratio, *CI* Confidence Interval

^*^Statistically significant variables at p < 0.05

^**^Statistically significant variables at p < 0.001

^a^Protestant, Catholic and traditional

^b^Southern nations, nationalities and peoples

## Discussion

The overall lifetime and current magnitude of psychoactive substance use among Woldia University students was 36.9% [95% CI 33.2, 40.7%] and 31.5% [95% CI 27.8, 35.1%], respectively. These findings were lower than similar studies done in Ethiopia. In Axum University, the overall lifetime magnitude of psychoactive substance use among students was 45.9%, and current magnitude of psychoactive substance use was 44.8% [13]. The overall lifetime and current magnitude of psychoactive substance use among Hawassa University students was 53.6 and 35.5%, respectively [14]. Moreover, in Debre Markos University the overall lifetime magnitude of psychoactive substance use among students was 48.4%, and the current magnitude was 46.3% [15]. The findings were also lower than the findings at Kenya where the lifetime prevalence of substance use was 69.8% [21]. This might be due to the difference in the regulations of particular universities, accessibility of psychoactive substances and nearby community culture.

The lifetime and current prevalence of khat chewing among Woldia University students were 13 and 10.4%, respectively. The lifetime prevalence of khat chewing was consistent with the findings at Addis Ababa University [12]. However, it was lower than the finding at Axum University where about 29% of the study participants had chewed khat in their lifetime, and nearly 28% were current khat chewers [13]. It was also lower than the findings at Bahir Dar University [17].

The lifetime and current prevalence of alcohol drinking were 33.1 and 27.9%, respectively. This was similar with the findings indicated at Northern Ethiopia [13]. But it was lower than the finding at Kenya, in which about 52% of the study students had drunk alcohol in their lifetime, and about 51% were current alcohol drinkers [21].

The lifetime and current prevalence of cigarette smoking were 7.9 and 6.4%, respectively. This was relatively consistent with the findings at Axum University [13].The current prevalence of cigarette smoking in Woldia University is higher than the finding at Addis Ababa University [12]. On the other hand, a cross sectional study on secondary school students in Zimbabwe revealed higher prevalence of cigarette smoking [22].

This study showed that males were more likely to chew khat, and to drink alcohol as compared to females in Woldia University. This finding was consistent with what has been found in Debre Markos and Haramaya Universities [15, 16]. In addition, being male was associated with alcohol use in the last 12 months in Addis Ababa University and Kenya [12, 21]. It was also associated with khat chewing in other studies [5, 6, 13, 17, 23].

Students who were Muslims and Oromo ethnic group were more likely to chew khat compared to students who were Ethiopian Orthodox Christians. This is consistent with other studies in Ethiopia [5, 6, 12, 13]. This could be explained in such a way that cultivation of khat is widely practiced in Oromia region of Ethiopia. On the other hand, alcohol drinking was lower among Muslims in Woldia University. This is supported with the findings of similar studies in Ethiopia [12, 16].

Compared to first year students, third year students were more likely to chew khat. Similarly, in Haramaya University third year students were more likely to use substances than other students [16]. This could be explained in such a way that students in later campus life might be stressed so that they might tend to use substances.

Students who had ever drunk alcohol and ever smoked cigarette were more likely to chew khat. This was consistent with a study done in Kenya [24]. In Addis Ababa and Mekelle Universities, ever use of cigarette was positively associated with khat chewing [12, 25]. Alternatively, khat chewing within the past 12 months was associated with increased odds of alcohol consumption [12]. Furthermore, ever khat chewing and alcohol drinking were associated with increased odds of cigarette smoking in Woldia University. Similarly, in Zimbabwe students who drink alcohol were more likely to smoke cigarette [22].

High amount of pocket money was associated with increased probability of using psychoactive substances in Woldia University. In Ethiopia, the middle and richest wealth quintiles were more likely to chew khat as compared to the poorest category [5]. Similarly, students who had more pocket money were more likely to use psychoactive substances as compared to those who received relatively less amounts [14, 26]. Furthermore, adolescents who received more pocket money in a month were more likely to be smokers [27]. This could be explained in such a way that Students who get access to a lot of money may be tempted to buy psychoactive substances.

The study could be subjected to the following limitations. First, since this was cross-sectional study it is unable to establish causality between independent and dependent variables. Second, the data were collected based on self-report of the study participants and may be subjected to recall and social desirability biases. Finally, since the study involved only university students, findings from this study may not be generalized to the whole young population. Despite of these limitations, the findings indicate a need to educate university students regarding the adverse effects of psychoactive substances use.

## Conclusion and recommendation

The overall lifetime prevalence of substance use among university students is 36.9%. In this study 33.1, 13 and 7.9% ever drunk alcohol, chewed khat and smoked cigarette, respectively. Majority (59.1%) of the students were introduced to psychoactive substance use by peer friends, and about 66% of the study students reported substances use to relax with friends. Average monthly pocket money, ethnicity and religion were also correlates of substances use. Therefore, the Ethiopian Ministry of Education needs to integrate topics about substance use in the university curriculum. Furthermore, to tackle substance use, emphasis should be given on educating and enlightening students on proper use of pocket money, establishing and strengthening peer educators in the university, and integrating health education and information on substance use in religious institutions.
